# The putative K^+^ channel subunit AtKCO3 forms stable dimers in *Arabidopsis*

**DOI:** 10.3389/fpls.2012.00251

**Published:** 2012-11-12

**Authors:** Alessandra Rocchetti, Tripti Sharma, Camilla Wulfetange, Joachim Scholz-Starke, Alexandra Grippa, Armando Carpaneto, Ingo Dreyer, Alessandro Vitale, Katrin Czempinski, Emanuela Pedrazzini

**Affiliations:** ^1^Istituto di Biologia e Biotecnologia Agraria, Consiglio Nazionale delle RicercheMilano, Italy; ^2^Institute of Biochemistry and Biology, University of PotsdamPotsdam-Golm, Germany; ^3^Istituto di Biofisica, Consiglio Nazionale delle RicercheGenova, Italy; ^4^Centro de Biotecnología y Genómica de Plantas, Universidad Politécnica de MadridMadrid, Spain

**Keywords:** *Arabidopsis*, membrane proteins, potassium channels, protein assembly, tonoplast

## Abstract

The permeation pore of K^+^ channels is formed by four copies of the pore domain. AtKCO3 is the only putative voltage-independent K^+^ channel subunit of *Arabidopsis thaliana* with a single pore domain. KCO3-like proteins recently emerged in evolution and, to date, have been found only in the genus *Arabidopsis* (*A. thaliana* and *A. lyrata*). We show that the absence of KCO3 does not cause marked changes in growth under various conditions. Only under osmotic stress we observed reduced root growth of the *kco3-1* null-allele line. This phenotype was complemented by expressing a KCO3 mutant with an inactive pore, indicating that the function of KCO3 under osmotic stress does not depend on its direct ability to transport ions. Constitutively overexpressed AtKCO3 or AtKCO3::GFP are efficiently sorted to the tonoplast indicating that the protein is approved by the endoplasmic reticulum quality control. However, vacuoles isolated from transgenic plants do not have significant alterations in current density. Consistently, both AtKCO3 and AtKCO3::GFP are detected as homodimers upon velocity gradient centrifugation, an assembly state that would not allow for activity. We conclude that if AtKCO3 ever functions as a K^+^ channel, active tetramers are held by particularly weak interactions, are formed only in unknown specific conditions and may require partner proteins.

## INTRODUCTION

Homeostasis of potassium, the most abundant cation of plants, is maintained by the activity of transporters and channels of the plasma membrane and the tonoplast (Dreyer and Uozumi, 2011). *Arabidopsis thaliana* contains 15 genes encoding K^+^ channel subunits. Among these are the five members (*At*TPK1–5) of the voltage-independent, tandem-pore family (Voelker et al., 2010). The key signature of K^+^ channels is the pore (P) domain which must be present in four copies in an active channel complex. Tandem-pore channels are therefore expected to be dimers. Consistently, fluorescence resonance energy transfer (FRET) and bimolecular fluorescence complementation (BiFC) experiments conducted on *At*TPK1 or *At*TPK5 in transiently transformed plant cells strongly support the existence of homodimers (Voelker et al., 2006). Stable heteromeric interactions between different *At*TPK members were instead not detected (Voelker et al., 2006). The assembly of *At*TPK1 into stable dimers has been directly confirmed in transgenic *Arabidopsis* constitutively expressing an AtTPK1::GFP fusion (Maîtrejean et al., 2011). An additional polypeptide with one pore domain only, *A*tKCO3 (locus AT5G46360), is most closely related to *At*TPK2 and believed to have originated from gene duplication followed by a partial deletion event (Marcel et al., 2010; Voelker et al., 2010). KCO3-like proteins have only been identified in the genus *Arabidopsis* (*A. thaliana* and *A. lyrata*), so far. They were not found in other known plant genomes (Gomez-Porras et al., 2012). Upon transient expression in protoplasts from *Arabidopsis* cell cultures, *At*KCO3::GFP localizes at the tonoplast, like similar fusions of *At*TPK1, 2, 3, and 5 (Voelker et al., 2006). Homomeric *At*KCO3 interactions have been detected by FRET and BiFC (Voelker et al., 2006) but it is not known whether the polypeptide forms tetramers, a prerequisite for activity in the case of this one-pore subunit. Here we have applied a number of experimental approaches in order to get insights on the assembly state and possible function of *At*KCO3.

## MATERIALS AND METHODS

### PLANT GENOTYPING AND ANALYSIS

The *A. thaliana* knock-out mutant *kco3-1* (Salk_096038) was ordered from the Salk Institute. Genomic DNA was extracted from frozen leaves with 1 ml of CTAB extraction buffer (0.8% CTAB, 0.14 M sorbitol, 0.22 mM Tris–HCl, pH 6, 0.022 mM EDTA, 0.8 M NaCl, 1% *N*-Lauroylsarcosine). Confirmation of the T-DNA insertion in *kco3-1* was done using the following primers: 5′GCGTGGACCGCTTGCTGCAACT3′ (T-DNA-LB), 5′CACGA-TTTCTATGCCAATGACTCCATCGG3′ (KCO3-fwd), 5′AAAAA-GAGCTCTTAAACTGGTTCAACTATATCC3′ (KCO3-rev).

For phenotypic analysis, seeds were plated on MS media supplemented with 3% sucrose in axenic condition. One-week-old seedlings were transferred to media containing the appropriate solute for the growth test in different abiotic stress conditions and were vertically grown in 16 h day/8 h night conditions. Different potassium concentration: seedlings were transferred on K^+^ deficient medium [2.5 mM NaNO_3_, 2.5 mM Ca(NO_3_)_2_, 2 mM NH_4_(H_2_PO_4_), 2 mM MgSO_4_, 0.1 mM FeNaEDTA, 25 µM CaCl_2_, 25 µM H_3_BO_3_, 2 µM ZnSO_4_, 2 µM MnSO_4_, 0.5 µM CuSO_4_, 0.2 µM Na_2_MoO_4_, 0.01 µM CoCl_2_, 1% sucrose, pH 5.7; jellified with 0.8% Phytagel (Sigma Aldrich)] supplemented with low (100 µM) or high (2.5 mM) K^+^; salt stress: 75 mM NaCl; osmotic stress: 100 mM mannitol. Oxidative stress: 15 days after sowing the seedlings were moved to liquid MS media with 10 mM H_2_O_2_. For mock treatment, plants were transferred to liquid MS media. Plants were grown on a shaker for 5 days, with daily change of media.

### GENERATION OF TRANSGENIC PLANTS

Plasmid DNA required for sequencing purposes was prepared using Qiagen columns (Qiagen, Hilden, Germany). Sequence determinations were performed by MWG-Biotech (Ebersberg, Germany) and Replicon (Berlin, Germany). For sequence analysis the BLAST server at the National Center of Biological Information (NCBI, Bethesda, USA), or the University of Wisconsin GCG software package, version 8 (Devereux et al., 1984) were used. Either Pfu polymerase (Stratagene, Heidelberg, Germany) or Taq polymerase (Gibco BRL, Eggenstein, Germany) was employed for PCR. All PCR-derived fragments were sequenced to ensure the absence of amplification errors.

**FIGURE 1:**
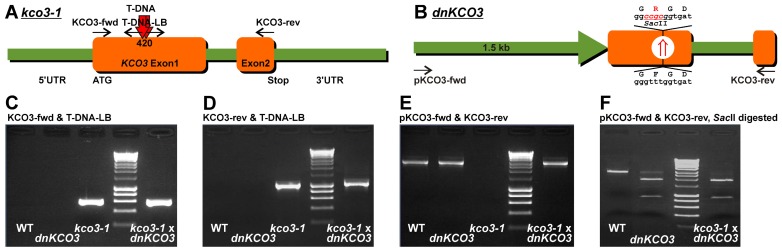
**Genotyping of *kco3-1* null-allele, *dnKCO3* and *kco3-1* × *dnKCO3* mutant plants. (A)** Schematic representation of the Salk_96038 T-DNA insertion line that contains two head to head T-DNA insertions at position 420 in the first exon of the KCO3 gene. **(B)** Schematic representation of *dnKCO3* mutant, where, in the first exon of the *KCO3* gene, a dominant negative mutation has been created by mutating the GFGD motif to GRGD. Additionally, a recognition site of the restriction enzyme *Sac*II has been inserted. **(C,D)** PCR was performed on genomic DNA with the indicated primer sets specific for T-DNA amplification. Amplification was attained in case of *kco3-1* and *kco3-1 *×* dnKCO3* while the wild-type and *dnKCO3* does not show any amplification product with the T-DNA primer sets. **(E)** PCR was performed with the indicated gene specific primer pair. Amplification can be observed in Col-0 wild-type, *dnKCO3* and *kco3-1 *×* dnKCO3*. No amplification product is detectable in the homozygous T-DNA insertion line (*kco3-1*). **(F)** *Sac*II restriction digestion of the PCR products obtained by amplification with the gene specific primer pair. PCR product from Col-0 wild-type was not digested. The amplification product from the *dnKCO3* mutant was digested but some undigested product can also be seen. The PCR product from *kco3-1 *× *dnKCO3* shows complete digestion.

To produce *dnKCO3* transgenic plants, site directed mutagenesis was performed on the *KCO3* gene to insert dominant negative mutation F141R. PCR product was digested with *Sal*I–*Sac*I and inserted in *Sal*I–*Sac*I digested pBS generating pBS-dnKCO3. To generate pBIBhygro-dnKCO3, amplification was done on pBS-dnKCO3 with pKCO3-fwd (5′ATTTAGTCGACACACATCACAA-CATGATTGAAGATGACAATG3′) and KCO3-rev primers (**Figure 1B**). Amplification product was double digested with *Sal*I–*Sac*I and cloned in *Sal*I–*Sac*I digested pBibHygro. Positive clones were further confirmed by sequencing and transformed in *Agrobacterium tumefaciens* strain GV1301. The positive *Agrobacterium* clones were detected through PCR with gene-specific primers on mini preparation of DNA from *Agrobacterium*. Over-night grown culture of *Agrobacterium* was infiltrated in Col-0 wild-type plants through floral dip method. Seeds from infiltrated plants were screened on hygromycin-containing medium to select transgenic plants. Seedlings surviving on hygromycin-containing medium were used for genotyping to detect the presence of transformed transgene.

To generate *kco3-1* × *dnKCO3* transgenic plants, homozygous lines of *dnKCO3* were crossed with *kco3-1* null-allele (Salk_96038) mutant. The seeds obtained from the crossed plants were then screened to procure dominant-negative knockout mutant plants of KCO3. These *kco3-1* × *dnKCO3* were grown again in the next generation under self-fertilization condition. Seeds thus inherited after self-cross were screened by performing PCR reactions to obtain homozygous *kco3-1* × *dnKCO3*.

AtKCO3 cDNA clones were isolated from cDNA preparations of *A. thaliana* seedlings (C24 ecotype) using primers 5′CAACAACAAGGACCCATTACACC3′ (KCO3.seq) and 5′CCACTGCCATCTTCAATCATG3′ (KCO3.rev). Fragments corresponding to the *AtKCO3* cDNA were subcloned into pPCRII (Stratagene, Heidelberg, Germany) giving rise to the plasmid pCRII-*KCO3*. Sequence determination was done for three clones as described above. Plasmid p35S-*KCO3* was generated by inserting the At*KCO3* cDNA (KpnI/blunt/EcoRV pCRII-*KCO3*) into SmaI site of pBinAR-Kan (Höfgen and Willmitzer, 1988). A C-terminal *GFP*-fusion construct was created using pBI-35S-10H-GFP-JFH1 (Hong et al., 1999). The *At*KCO3 cDNA was amplified using the primers 5′CGCGTCGACATGCCAATGACTCCATC-GG3′ (KCO3-FSI.seq) and 5′ATCACTAGTACAGAAGTTGCGG-TGGTTAAATCCAA3′ (KCO3-FSII.rev) and fused to *GFP* coding sequence via *Sal*I/*Xho*I*/Spe*I sites to generate p35S-*KCO3::GFP*.

Transgenic *Arabidopsis* plants expressing KCO3 or KCO3::GFP were generated by vacuum infiltration with *Agrobacterium tumefaciens* strain GV3101 transformed with the constructs p35S-*KCO3* or 35S-*KCO3::GFP*. Kanamycin-resistant plants (T0) were identified. Experiments were conducted using T3 or T4 plants.

### ANTIBODIES

The following antibodies were used in this study: rabbit polyclonal anti-GFP (1:1000 dilution, Invitrogen), rabbit polyclonal anti-endoplasmin/GRP94 (1:1,000 dilution; Klein et al., 2006), rabbit polyclonal anti-PiP2 (1:10,000 dilution; Santoni et al., 2003), or chicken polyclonal anti-γTIP raised against a synthetic peptide corresponding to the C-terminal nine amino acids of *Arabidopsis* γTIP (1:1,000 dilution, a gift from N. V. Raikhel).

An anti-KCO3 antiserum was raised against a synthetic peptide (NH_2_-SEFKNRLLFGSLPRC-COOH) located at the N-terminus of AtKCO3 and provided by Covalab (Villeurbanne, France). The total IgG fraction from immunized rabbit was then purified using protein A bead column. The affinity-purified antibodies were then subjected to immuno-affinity purification, passing through a resin-column containing the antigenic peptide. This affinity-purified rabbit polyclonal anti-KCO3 was used at 1:2,000 dilution.

### VACUOLE ISOLATION, PATCH-CLAMP RECORDINGS, AND DATA ANALYSIS

Mesophyll tissue of *Arabidopsis* plants was enzymatically digested for 30 min at 30°C. The enzyme solution contained 0.3% (w/v) cellulase R-10, 0.03% (w/v) pectolyase Y-23, 1 mM CaCl_2_, 500 mM sorbitol, 10 mM 2-(*N*-morpholino)ethanesulfonic acid (MES), pH 5.3. Protoplasts were washed twice and maintained in W5 solution (125 mM CaCl_2_, 154 mM NaCl, 5 mM KCl, 2 mM MES-KOH, pH 5.6; Yoo et al., 2007). Vacuoles were released by the addition of 10 volumes of a solution containing 100 mM malic acid, 160 mM 1,3-bis(tris(hydroxymethyl)methylamino)propane (BTP), 5 mM ethylene glycol-bis(2-aminoethylether)-*N*,*N*,*N*′,*N*′-tetraacetic acid (EGTA), 3 mM MgCl_2_, pH 7.5, adjusted to 450 mOsm with D-sorbitol. After settling of the vacuoles, the recording chamber was carefully perfused with fresh bath solution. Membrane currents of isolated vacuoles were recorded using the patch-clamp technique, as described elsewhere (Scholz-Starke et al., 2006; Gradogna et al., 2009). Trans-membrane voltages and ionic currents were controlled and monitored with an Axon 200-A current-voltage amplifier interfaced with a 16 bit AD/DA board (ITC-16 Instrutech, Elmont, NY, USA). A Macintosh personal computer running the Pulse program (Heka Electronic, Lambrecht, Germany) was used to generate the stimulation protocol and to acquire the current records. Current records were low-pass filtered at 200 Hz with a 4-pole Bessel filter Kemo VBF8 (Kemo, Beckenham, UK) and sampled at a frequency of 1 kHz. All patches with seal resistance lower than 3 GOhm were systematically discarded. Membrane capacitance (*C*_m_) and access resistance (*R*_a_) were estimated by using the compensation circuit of the amplifier; *R*_a_ varied from 6 to 10 MOhm and was not corrected, since the resulting voltage error did not exceed 5 mV. *C*_m_ and *R*_a_ were monitored throughout the experiment. No leak current subtraction procedure was applied. Patch pipettes had resistance values of 3.5–4 MOhm, when filled with pipette solution (vacuolar side) containing 200 mM KCl, 2 mM CaCl_2_, 2 mM MgCl_2_, 10 mM MES-KOH, pH 5.5. All bath solutions contained 100 mM KCl, 1 mM dithiothreitol (DTT), 5 mM Tris and additionally 1 mM EGTA for low-calcium conditions or 100 µM CaCl_2_ for high-calcium conditions. The pH value of the bath solutions was set either to pH 7.5 (by addition of HEPES) or to pH 6.5 (by addition of MES). The osmolarity of the pipette and bath solutions was adjusted to 500 and 540 mOsm, respectively, by the addition of D-sorbitol. Liquid junction voltages in our experimental conditions were smaller than 1 mV.

**Figure A1:**
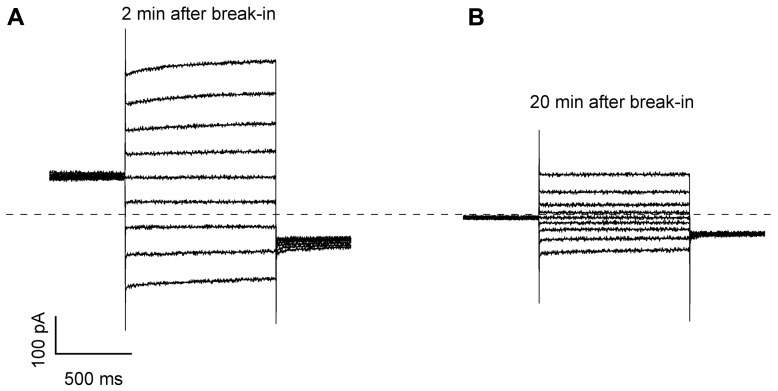
**Current traces recorded in whole-vacuole mode in vacuoles isolated from *Arabidopsis* mesophyll cells. (A)** Current traces recorded 2 min after achieving the whole-vacuole configuration in response to voltage steps from +80 to -80 mV, step -20 mV. The holding and tail voltages were 0 and -50 mV, respectively. The vacuole had a capacitance of 34 pF and derived from an *Arabidopsis* wild-type plant. **(B)** Current traces recorded from the same vacuole as in A, 20 min after the break-in.

Immediately after the establishment of the whole-vacuole configuration, current recordings reproducibly displayed background currents with negative reversal potentials (**Figure A1A**). These currents gradually decreased at all membrane potentials and finally stabilized at a slightly positive reversal potential after 15–30 min in the whole-vacuole configuration (**Figure A1B**), depending on the vacuole size. Only vacuoles which reached this point were considered for data analysis and successively exposed to other bath solutions by means of a gravity-driven perfusion system coupled to a peristaltic pump. Steady-state current amplitudes were normalized to the vacuolar membrane capacitance, which was on average 30 ± 3 pS (*n* = 22). Data are represented as mean values ± SEM (for the number *n* of vacuoles).

### TOTAL PROTEIN ANALYSIS

*Arabidopsis* wild-type or transgenic plants were grown in sterile conditions on half-concentrated MS media (Duchefa Biochemie) supplemented with 10 g/l sucrose and 0.8% (w/v) phyto agar (Duchefa Biochemie) at 23°C under a 16/8 h light/dark cycle. 3–6 weeks old leaves were homogenized in ice-cold homogenization buffer [200 mM NaCl, 1 mM EDTA, 0.2% Triton X-100, 2% 2-mercaptoethanol, 100 mM Tris–Cl pH 7.8, supplemented with Complete protease inhibitor cocktail (Roche)]. After centrifugation at 5,000 × *g* for 10 min at 4°C, the resulting supernatant was considered as the total protein extract and analyzed by SDS-PAGE followed by western blot, as follows. Samples were adjusted to 1% SDS, 0.3% 2-mercaptoethanol, 8.3% glycerol, 20 mM Tris–Cl, pH 8.6 and denatured by heating at 95°C for 4 min. SDS-PAGE was performed in 15% acrylamide gels using a Tris-glycine running buffer. After electrophoresis, proteins were transferred to nitrocellulose membrane (Protran, Whatman) using a Tris/glycine/methanol (25 mM/192 mM/ 20%, respectively) buffer in a wet electroblotting system (Trans-Blot^®^ Cell, Bio-Rad). We verified that even protein polymers larger than 600 kDa are efficiently transferred using this protocol. Molecular weight markers (Fermentas) were used as molecular mass markers.

### SUBCELLULAR FRACTIONATION

Total microsomes were prepared as follows: leaf tissues were homogenized in a medium containing 50 mM Tris-acetate (pH 7.5), 250 mM sorbitol, 2 mM EGTA, 2 mM MgCl_2_, 2 mM DTT supplemented with Complete. The homogenate was filtered and centrifuged at 10,000 × *g* for 10 min at 4°C. The supernatant was further centrifuged at 100,000 × *g* (r_av_) in a Beckman SW55Ti rotor (Beckman Instruments) for 30 min at 4°C. The resulting pellet, containing total microsomes, was resuspended in 10 mM Tricine-KOH (pH 7.5), 1 mM EGTA, 2 mM MgCl_2_, 5% (w/w) sucrose, and was loaded on a sucrose-density gradient (10.4 ml, 15–45% w/w sucrose in 10 mM Tricine-KOH (pH 7.5), 1 mM EGTA and 2 mM MgCl_2_), centrifuged at 77,000 × *g* (r_av_) for 19 h in a Beckman SW40 rotor and collected in 0.6-ml fractions. Equal volumes of each fraction were analyzed by SDS-PAGE followed by western blot with the appropriate antibody.

### VELOCITY SUCROSE GRADIENT CENTRIFUGATION

*Arabidopsis* leaves (3–6 weeks old) were homogenized in ice-cold buffer containing 40 mM KCl or 200 mM NaCl, 0.2% Triton X-100, 50 mM Tris–Cl pH 7.8 (2 ml/g of leaf tissue), supplemented with Complete. Lysate was cleared by centrifugation at 700 × *g* for 10 min, loaded on a linear 5–25% (w/v) sucrose gradient (20 mM KCl or 150 mM NaCl, 0.1% Triton X-100, 25 mM Tris–Cl, pH 7.5) and centrifuged at 200,000 × *g* (r_av_) for 25 h at 4°C in a Beckman SW40 rotor. An additional gradient was loaded with a mixture of molecular mass markers containing 200 µg each of cytochrome *c* (12.4 kDa), ovalbumin (43 kDa), bovine serum albumin (67 kDa), aldolase (161 kDa), and catalase (232 kDa). Equal volumes of each fraction were analyzed by SDS-PAGE followed by western blot with the appropriate antibody.

### MICROSCOPY

Epifluorescence microscopy on plant tissues was performed using a Zeiss Axiovert 200 microscope (Carl Zeiss) equipped for epifluorescence, followed by the collection of optical sections using Zeiss Apotome and Axiovision 4.1 software. Figures were assembled with Adobe Photoshop 10.0.

## RESULTS

### THE ABSENCE OF KCO3 CAUSES SLIGHT GROWTH DEFECTS UNDER OSMOTIC STRESS

**FIGURE 2:**
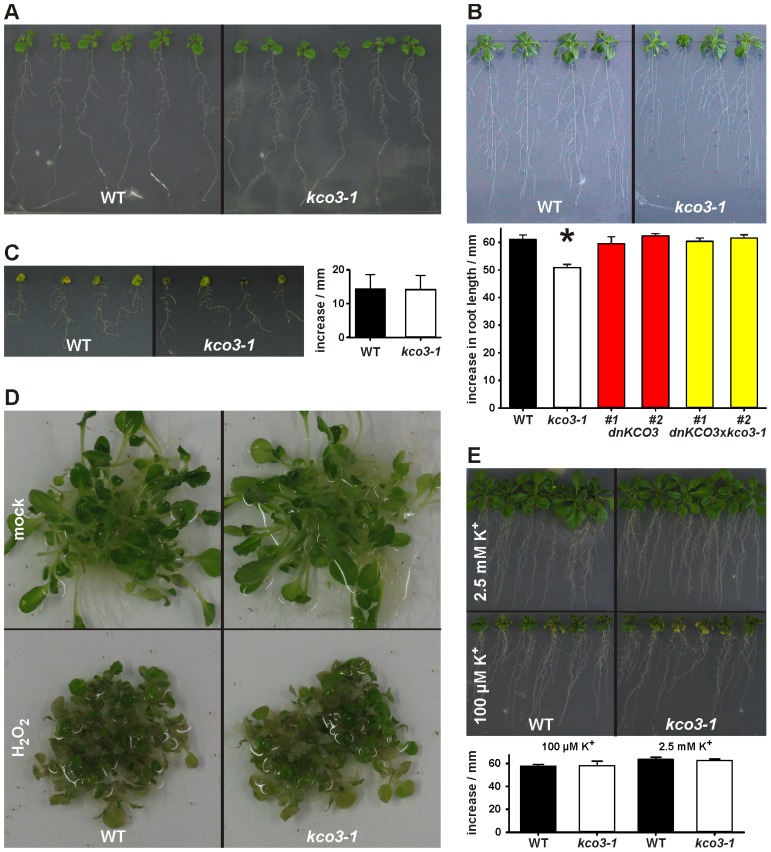
**Phenotypic analysis of kco3-1. (A)** Growth on MS medium supplemented with 3% sucrose. The wild-type and the *kco3-1* knock-out mutant do not show significant differences in shoot development and root length. Figure is representative of three independent experiments. **(B)** Osmotic stress. The figure is representative of three independent experiments. Lower panel: increase in root length after 15 days. The values shown are mean of six repeats ± SD (indicated by error bars). Data were analyzed using Student’s *t*-test. The values obtained for *kco3-1* are significantly different from those of the WT control, *dnKCO3* and *kco3-1 *× *dnKCO3* plants (*P* < 0.001). **(C)** Salt stress. Growth of Col-0 and *kco3-1* plants was severely hampered by the presence of NaCl. The figure is representative for three independent experiments. Right panel: increase in root length after 15 days. The values shown are mean of six repeats ± SD (indicated by error bars). Student’s *t*-test revealed that Col-0 and *kco3-1* are not significantly different (*P* > 0.1). **(D)** Oxidative stress. After five days of H_2_O_2_ stress, Col-0 and *kco3-1* plants showed symptoms such as bleaching of leaves and plant decay. The figure is representative for two independent experiments. **(E)** K^+^ deficient and K^+^ sufficient medium. Lower panel: increase in root length after 15 days was plotted as means ± SD (indicated by error bars) of six repeats. Student’s *t*-test did not reveal any significant difference between Col-0 and *kco3-1* (*P* > 0.1).

Inactivation of the KCO3 gene could provide insights on the function of the protein. The Salk_96038 T-DNA insertion mutant was analyzed using T-DNA left border primer and *KCO3*-specific forward and reverse primers. Amplification was obtained with both primer sets and showed two head-to-head T-DNA insertions at position 420 bp within *KCO3* exon 1, as shown schematically in **Figure 1**. End-point PCR, with a couple of *KCO3*-specific primers, performed on genomic DNA from *Arabidopsis* T-DNA insertion line did not give rise to any product (**Figure 1E**, *kco3-1*), while a fragment of the correct length was obtained from wild-type plant DNA (**Figure 1E**, wt). The insertion line is therefore a full loss of function mutant and was termed *kco3-1*. Col-0 and *kco3-1 *seeds were sown on MS medium with 3% sucrose. Seven days after sowing, the seedlings were grown for 15 days on MS medium under long day conditions. No significant (Student’s *t*-test) differences were observed in root development, leaf number, and leaf size (**Figure 2A**). The response to osmotic stress or salt stress was tested by supplementing the MS medium with 100 mM mannitol or 75 mM NaCl. Mannitol caused a small but significant decrease of root growth in *kco3-1* plantlets with respect to Col-0 (**Figure 2B**). This phenotype could be complemented by expressing a dominant-negative KCO3-mutant under control of the AtKCO3 promoter in the *kco3-1* background (**Figure 2B**; *dnKCO3 *× *kco3-1*). Expressing the dominant-negative mutant in the wild-type background had no beneficial effect for the plant (**Figure 2B**; *dnKCO3*). Upon NaCl treatment no difference was observed (**Figure 2C**). The effect of oxidative stress was tested by growing plantlets for 5 days in the presence of 10 mM H_2_O_2_. Both Col-0 and *kco3-1* showed similar symptoms of bleaching and plant decay (**Figure 2D**). Finally, growth in medium supplemented with 100 µM K^+^ (K^+^ deficient) or 2.5 mM K^+^ (K^+^ sufficient) was analyzed (**Figure 2E**). No significant differences were observed in the mean values for increase in root length.

Altogether, these analyses indicate that the absence of KCO3 causes small defects in the early stages of plant growth under osmotic stress. These defects could be complemented by a dominant-negative mutant of KCO3.

### KCO3 AND KCO3::GFP CONSTITUTIVELY EXPRESSED IN *ARABIDOPSIS* TRANSGENIC PLANTS LOCALIZE AT THE TONOPLAST

**FIGURE 3:**
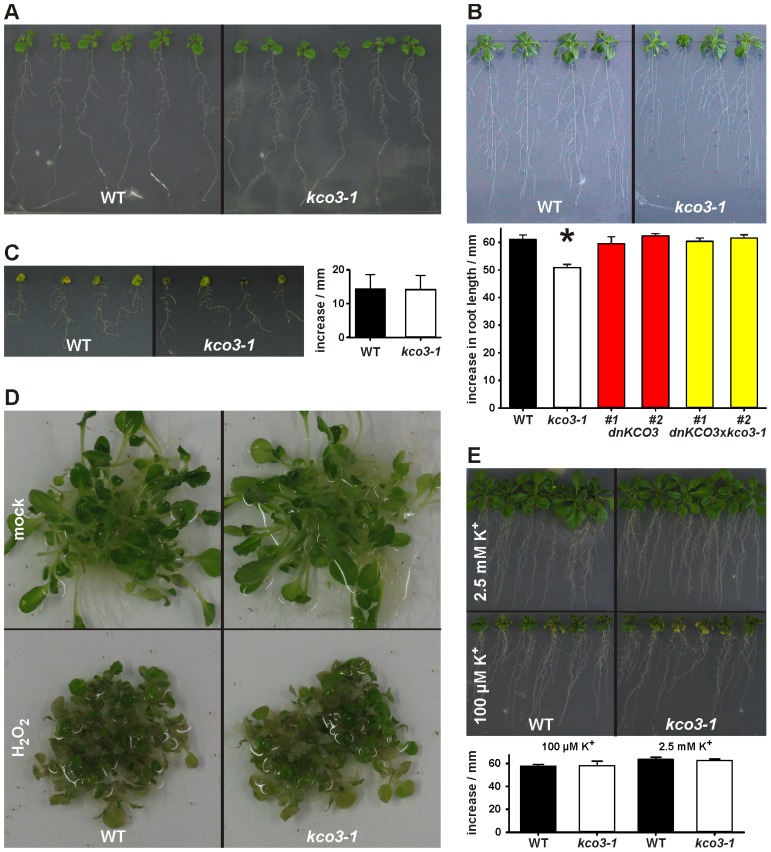
**Selection of *Arabidopsis* plants overexpressing KCO3::GFP and KCO3. (A)** Equal amounts of leaf extracts (50 µg of total protein) from wild-type plants (Co) and independent transgenic *Arabidopsis* plants expressing KCO3::GFP (plants 1–11) were subjected to SDS-PAGE and western blot analysis with anti-GFP antiserum. The positions of the entire fusion protein and of free GFP are indicated. **(B)** Leaf extracts from one wild-type plant (lane 1) and transgenic plant *n*° 6 of panel A (lane 2) were analyzed by western blot using anti-KCO3 antibodies. The positions of monomers (arrowhead) and putative dimers (circle) are indicated. **(C)** Equal amounts (50 µg of total protein) of leaf extracts from wild-type (Co) or independent transgenic *Arabidopsis* plants overexpressing KCO3 (plants 2–7) were analyzed by western blot with anti-KCO3 antiserum. The positions of KCO3 (arrow) and an unspecific, cross-reacting polypeptide (asterisk) are indicated. In each panel, numbers on the left indicate the position and size (in kDa) of molecular mass markers.

Endogenous expression levels of KCO3 are very low (Schönknecht et al., 2002, and see eFP developmental map: http://bbc.botany.utoronto.ca/efp/cgi-bin/efpWeb.cgi), hampering biochemical and cell biology studies. *Arabidopsis* transgenic plants expressing KCO3::GFP under the constitutive CaMV 35S promoter were therefore produced. Transgenic lines, selected on the basis of their kanamycin resistance, were tested for KCO3::GFP accumulation by western blot analysis of leaf extracts using anti-GFP antiserum (**Figure 3A**). Extracts from wild-type plants (**Figure 3A**, Co) did not react with the antiserum. A polypeptide with apparent molecular mass around 60 kDa was specifically detected in the kanamycin-resistant plants. This is in good agreement with the predicted mass of the fusion between KCO3 (29 kDa) and GFP (27 kDa), indicating that the 60 kDa polypeptide is intact or nearly intact KCO3::GFP (**Figure 3A**, KCO3::GFP). An additional polypeptide around 25 kDa was also detected in some of the transgenic plants, often when accumulation of KCO3::GFP was higher. Its molecular mass suggests that it corresponds to the entire or nearly entire GFP sequence, released from fusion protein (**Figure 3A**, plants 1, 2, 4, 11 – free GFP). Accumulation of KCO3::GFP in T1 and T2 generations was highly variable but it became stable in a number of T3 plants and generations. These plants did not show evident morphological and developmental differences compared to the wild-type. We reasoned that the detection of KCO3::GFP using the anti-GFP antiserum could limit our studies, because this does not allow to follow the destiny of KCO3 once GFP has been released. We therefore produced a specific antiserum against KCO3 (anti-KCO3), using as antigen 15 amino acids near the N-terminal end (residues 6–20). To test the specificity of this antiserum, equal amounts of total leaf homogenate from KCO3::GFP transgenic or control plants were subjected to SDS-PAGE and western blot with anti-GFP or the immuno-affinity purified anti-KCO3 antiserum (**Figure 3B**). No bands were visible in the control sample, consistent with the known very low endogenous expression level of KCO3. In the transgenic extract the anti-KCO3 antiserum specifically recognized two polypeptides around 60 and 120 kDa (**Figure 3B**, lane 4, arrowhead and empty circle, respectively) and, as expected, did not recognize the 25 kDa fragment. The 60 kDa polypeptide corresponds to the entire KCO3::GFP fusion also detected by anti-GFP. At this stage of investigation the 120 kDa polypeptide was tentatively identified as a minor proportion of KCO3 polypeptides not completely denatured and still assembled into dimers. The ratio between these putative dimers and monomers was variable in different experiments. We tested different extraction and denaturation procedures, but we were unable to determine conditions in which such a variability could be avoided.

Having confirmed the ability of the antiserum to detect KCO3, transgenic *Arabidopsis* overexpressing KCO3 under the CaMV 35S promoter were prepared. Leaf homogenates were analyzed by SDS-PAGE and western blot with the anti-KCO3 antiserum. A polypeptide with apparent molecular mass around 30 kDa, consistent with the 29 kDa calculated mass of KCO3, was detected in the transgenic lines but not in control plants (**Figure 3C**, plants 2–7 and Co, respectively). A polypeptide around 20 kDa was also detected in both control and KCO3 plants (**Figure 3C**, asterisk); however, this was not related to KCO3, because it was also recognized by the pre-immune serum (not shown). We propagated line 6 from T3 generation, but also in T4 the accumulation levels of KCO3 were variable. We were unable to obtain a line giving constant KCO3 levels in the progeny, possibly because of transgene silencing.

**FIGURE 4:**
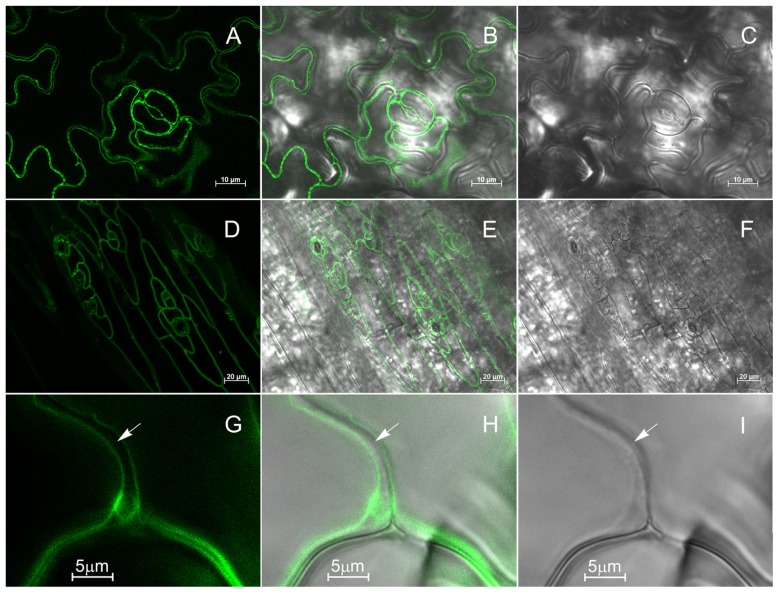
**KCO3::GFP is located at the tonoplast.** Leaf epidermis **(A–C, G–I)** or hypocotyl **(D–F)** from KCO3::GFP transgenic plant were analyzed by epifluorescence microscopy. **(A,D,G)**: GFP fluorescence; **(C,F,I)**: brightfield; **(B,E,H)**: Merge of fluorescence and brightfield. The arrow indicates the cell surface.

**FIGURE 5:**
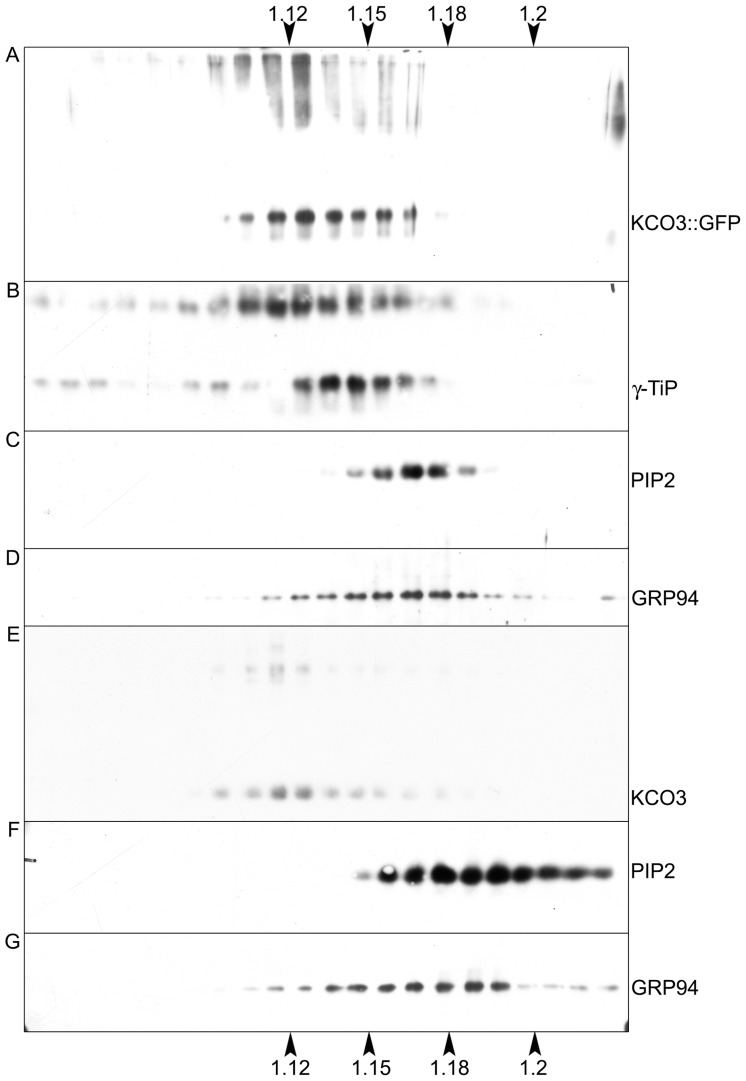
**KCO3::GFP and KCO3 co-fractionate with a tonoplast marker in isopycnic gradients.** Young leaves from transgenic *Arabidopsis* plants expressing KCO3::GFP **(A–D)** or KCO3 **(E–G)** were homogenized in the presence of sucrose and the absence of detergent. Microsomes were pelleted and further separated by centrifugation on isopycnic sucrose gradient. Proteins in each fraction (1/10 of fraction volume) were analyzed by SDS-PAGE and western blot with antibodies against KCO3 (to detect KCO3::GFP or KCO3), γTIP (tonoplast marker), PIP2 (plasma membrane marker) or endoplasmin (GRP94, endoplasmic reticulum marker), as indicated at the right of each panel. Top of each gradient is at left; numbers on top or bottom of the figure indicate fraction density (g/ml).

The subcellular localization of KCO3::GFP was analyzed by epifluorescence microscopy in leaf and hypocotyl tissues (**Figure 4**). In leaf epidermal cells, GFP fluorescence was clearly detected on the tonoplast (**Figures 4A–C**). In different cells, the GFP fluorescence appeared either continuous or irregularly distributed on the tonoplast, with several highly fluorescent “spots.” No colocalization with the plasma membrane was observed when the GFP and the brightfield images were superimposed (**Figures 4G–I**). The tonoplast localization was also observed in hypocotyl cells (**Figures 4D–F**). The results were confirmed by subcellular fractionation followed by western blot. Total microsomes were prepared from KCO3::GFP or KCO3 leaf homogenates, loaded onto a continuous sucrose gradient and subjected to isopycnic ultracentrifugation (**Figure 5**). The distributions of KCO3::GFP, KCO3, and the tonoplast aquaporin γTIP were similar and they markedly differed from those of the plasma membrane aquaporin PIP2 or the endoplasmic reticulum marker endoplasmin/GRP94. γTIP monomers have a calculated molecular mass around 20 kDa, but a relevant proportion of the protein is detected as oligomers, most probably corresponding to dimers; incomplete denaturation, resulting in the detection of dimers and tetramers upon SDS-PAGE is a common characteristic of aquaporins (Kammerloher et al., 1994; Karlsson et al., 2000). It can be concluded that both KCO3::GFP and KCO3 localize at the tonoplast when overexpressed in *Arabidopsis* transgenic plants.

### VACUOLES FROM WILD-TYPE AND KCO3-OVEREXPRESSING PLANTS DO NOT SHOW SIGNIFICANTLY DIFFERENT CURRENT DENSITIES

In order to investigate the functional properties of KCO3, we performed patch-clamp recordings on whole-vacuoles isolated from leaves of KCO3-overexpressing plants. Experiments were designed in a way to minimize background currents. The activity of the major cationic ion channels at resting and elevated cytosolic [Ca^2+^] – the Fast Vacuolar (FV) and the Slow Vacuolar (SV) channel, respectively (Hedrich and Neher, 1987; Allen and Sanders, 1996) – was greatly reduced by divalent cations, i.e., calcium (Tikhonova et al., 1997; Dadacz-Narloch et al., 2011) and magnesium (Pei et al., 1999; Pottosin et al., 2004), which were present in the pipette solution at 2 mM concentration. Preliminary experiments had revealed normal FV- and SV-type currents in vacuoles from *kco3-1* mutant plants (data not shown). These data, together with the presence of a K^+^ channel signature within the KCO3 protein sequence, argue against a possible participation of KCO3 in the pore formation of these non-selective cation channels.

**FIGURE 6:**
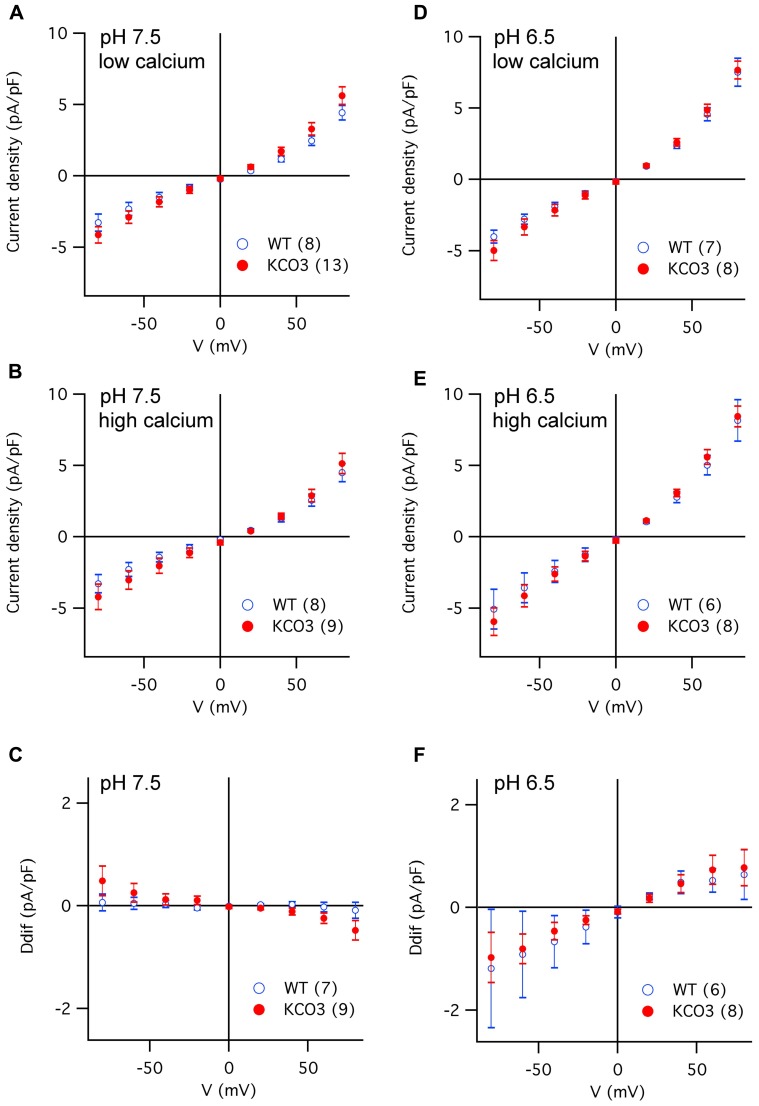
**Current density versus voltage characteristics recorded in different experimental conditions from vacuoles of *Arabidopsis* wild-type and KCO3-overexpressing plants. (A–C)** Current density–voltage relationships recorded in the whole-vacuole configuration (see **Figure A1**) from vacuoles of *Arabidopsis* wild-type (open blue symbols) and KCO3-overexpressing plants (filled red symbols). Bath solutions of pH 7.5 contained either low calcium **(A)** or high calcium **(B)**. The current density difference (Ddif) between high and low calcium conditions was calculated for individual vacuoles and averaged **(C)**. **(D,F)** As in **(A–C)**, but in bath solutions of pH 6.5, in low calcium **(D)**, in high calcium **(E)**, difference (**F**; Ddif). In each panel, the number of WT or KCO3 vacuoles analyzed is given in brackets.

The K^+^ channel signature in the pore loop and two calcium-binding EF hand motifs present in the KCO3 sequence are features shared by TPK1, which has been previously shown to encode a Ca^2+^-activated K^+^-selective conductance in the vacuolar membrane (VK channel; Gobert et al., 2007; Latz et al., 2007). Furthermore, the activity of VK channels is stimulated by acidic cytosolic pH (Allen et al., 1998; Gobert et al., 2007). Consequently, we probed KCO3 activity by recording membrane currents from isolated vacuoles successively exposed to four different experimental conditions, varying the cytosolic Ca^2+^ concentration (low versus high Ca^2+^) at two cytosolic pH values (pH_cyt_ 7.5 and pH_cyt_ 6.5). As summarized in **Figure 6**, the current densities of KCO3-overexpressing vacuoles were almost identical to those determined for wild-type vacuoles at either pH_cyt_. Increases of cytosolic [Ca^2+^] generally caused only minor changes in the current amplitudes: the slight decrease at pH_cyt_ 7.5 (**Figure 6C**) was possibly due to a further reduction of residual FV channel activity, while the small increase at pH_cyt_ 6.5 (**Figure 6F**) may correspond to the activation of a small population of VK channels.

In summary, these data strongly suggest that KCO3, present in the vacuolar membrane of overexpressing plants, does not constitute a functional ion channel in our experimental conditions.

### KCO3 AND KCO3::GFP ARE DETECTED AS DIMERS

**FIGURE 7:**
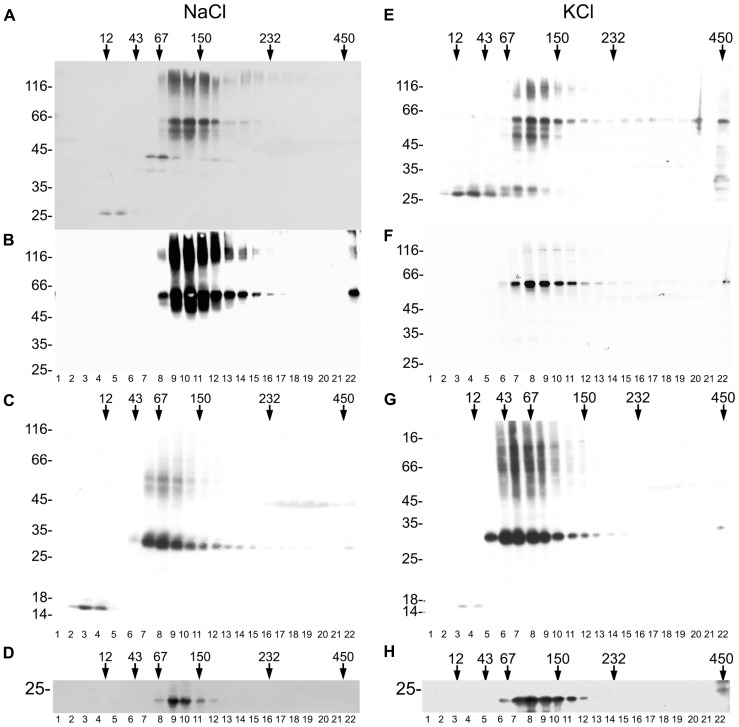
**KCO3::GFP and KCO3 are dimers.** Leaves from KCO3::GFP **(A,B,D–F,H)** or KCO3 **(C,G)** transgenic *Arabidopsis* plants were homogenized in the presence of non-ionic detergent and either 200 mM NaCl **(A–D)** or 40 mM KCl **(E–H)**. The homogenates were then subjected to sedimentation velocity centrifugation on a continuous 5–25% (w/v) sucrose gradient. One-tenth of the total volume of each gradient fraction were analyzed by SDS-PAGE and western blot using anti-GFP antiserum to detect KCO3::GFP **(A,E)**, anti-KCO3 antiserum to detect KCO3::GFP **(B,F)** or KCO3 **(C,G)**, anti-γTIP **(D,H)**. Top of each gradient is at left. Numbers on top indicate the position along the gradient and the molecular mass (in kDa) of sedimentation markers. Numbers at left indicate the positions of SDS-PAGE molecular mass markers.

The formation of a tetrameric complex is a prerequisite for ion channel activity in the case of the one-P subunit KCO3. We therefore investigated on the oligomerization state of KCO3 and KCO3::GFP. Total leaf homogenate from KCO3::GFP plants was fractionated by velocity sucrose gradient centrifugations in the presence of non-ionic detergent. Using this assay, we have previously confirmed that TPK1::GFP is a dimer, as expected (Maîtrejean et al., 2011). Equal volumes of fractions were analyzed by western blot with anti-GFP (**Figure 7A**) or anti-KCO3 (**Figure 7B**) antisera. KCO3::GFP sedimented slightly slower than the 150 kDa marker, indicating a dimer of the 60 kDa polypeptide. Each gradient fraction contains a similar ratio between polypeptides that are fully denatured upon SDS-PAGE and polypeptides that remain assembled into putative dimers (**Figures 7A,B**, fractions 9–12). The co-migration of the two forms along the gradient strongly suggests that the component around 120 kDa really represents dimers. No polypeptides recognized by anti-GFP or anti-KCO3 antisera peaked in the expected position of putative tetramers (their migration should be slightly faster than the 232 kDa marker). Free GFP released from the fusion protein migrated as a monomer and was recognized by the anti-GFP antiserum (**Figure 7**, compare **Figure 7A** and **Figure 7B**, fractions 4–5). This strongly suggests that the GFP moiety does not have a role in the dimerization of KCO3::GFP. The same behavior had been observed for free GFP released from TPK1::GFP (Maîtrejean et al., 2011). Partial proteolysis also seems to occur, as indicated by the additional bands detected below the major 66 kDa band.

To exclude that GFP appended at the C-terminal end of KCO3 impaired tetramerization, velocity gradient centrifugation analysis was performed on leaf extracts from KCO3 transgenic plants. Fractions were analyzed by western blot with anti-KCO3 antiserum. KCO3 sedimented slightly slower than the 67 kDa marker (**Figure 7C**, fractions 7–9), indicating that also the wild-type protein is a dimer in the conditions used in our analysis. We then tested whether the assembly grade of another multispanning tonoplast protein was preserved in our assay. Western blots of gradient fractions were therefore visualized with anti-γTIP antiserum: γTIP, which is a tetramer, peaked as expected between the 67 and 150 kDa markers (**Figure 7D**, fractions 9–10). We conclude that our experimental conditions preserve the correct assembly of another multispanning tonoplast protein.

It has been previously reported that tetramerization of the viral K^+^ channel Kcv was perturbed in the presence of a high concentration of sodium ions, but was maintained if 40 mM KCl was used instead of 200 mM NaCl (Pagliuca et al., 2007). We therefore analyzed the assembly state substituting NaCl with KCl in the homogenization and gradient buffers. Neither tetramers or monomers of KCO3::GFP and KCO3 could be detected, and the assembly state of γTIP was not altered as well (**Figures 7E–H**).

Therefore, stable KCO3 or KCO3::GFP homodimers are detected in experimental conditions in which the related TPK1 protein and the other multispanning tonoplast protein γTIP maintain their correct assembly properties. This indicates that KCO3 tetramers are either held together by weaker or very transient interactions, or are never formed.

## DISCUSSION

In this study, we applied different experimental approaches with the aim to shed light on the functional properties and physiological roles of AtKCO3, a putative potassium channel subunit in *A. thaliana*, which has remained enigmatic until now.

The features of the *kco3-1* null-allele mutant observed in the presence of mannitol indicate that under certain conditions KCO3 exhibits a small effect on root elongation. The absence of KCO3 could be compensated by a dominant-negative version of this subunit. This indicates that, whatever the function of KCO3, it does not depend on putative direct ability to transport ions. It is however clear that in most conditions no significant alteration in growth was detected.

Using transgenic plants expressing the *KCO3* gene or a *KCO3::GFP* fusion construct under the control of the constitutive CaMV 35S promoter, we confirmed and extended to stable transgenic *Arabidopsis* previous results from transient expression experiments (Voelker et al., 2006) on the tonoplast localization of KCO3. Patch-clamp recordings were therefore performed on vacuoles isolated from KCO3-overexpressing plants, in order to provide evidence of ion channel activity. In the design of these experiments, we started from the idea that KCO3 activity may be regulated, either positively or negatively, by cytosolic calcium via the canonical EF-hand motifs in the protein’s C-terminal domain. Previous studies have unequivocally established that AtTPK1, a well-characterized member of the tandem-pore family in *Arabidopsis*, encodes a Ca^2+^-activated K^+^-selective conductance in the vacuolar membrane, the so-called VK channel (Gobert et al., 2007; Latz et al., 2007). Conversely, our data set on KCO3-containing vacuoles did not reveal significant differences compared to background currents recorded from wild-type vacuoles. In similar working conditions, TPK1 activity was detected both in transformed yeast cells (Latz et al., 2007) and overexpressing *Arabidopsis* plants (Gobert et al., 2007).

The failure to obtain evidence for changes in current in the overexpressing plants prompted us to investigate on the KCO3 assembly status. To be active as a cation channel, KCO3 must either form homotetramers or contribute to the formation of a hetero-oligomer with four P-domains in total. However, we have only been able to detect homodimers of KCO3 or KCO3::GFP, in line with its evolutionary origin from dimeric TPK channels. The fact that both recombinant proteins, which have quite different molecular masses, migrate along velocity gradients at the positions expected for the corresponding homodimers rules out the possibility that the forms we detect are hetero-oligomers containing other unknown polypeptides. We have previously used this velocity centrifugation assay to study the assembly state of wild-type or different mutated forms of TPK1 fused to GFP (Maîtrejean et al., 2011). Constructs unable to traffic along the secretory pathway, and therefore with incorrect subunit interactions, could be distinguished by the assay from correctly assembled dimeric TPK1::GFP (Maîtrejean et al., 2011). This indicated that wrongly assembled forms of a tandem-pore channel are unable to traffic and that the incorrect interactions can be detected by the velocity centrifugation assay. The tonoplast localization of KCO3 and KCO3::GFP in transgenic *Arabidopsis* instead indicates that these proteins traffic along the secretory pathway and therefore are not recognized as defective proteins by the endoplasmic reticulum quality control.

The stable assembly of a dimeric form of a cation channel subunit with one P-domain is thus compatible with traffic, but it is clearly incompatible with activity. KCO3 is not the only *Arabidopsis* K^+^ channel that does not show activity. AtKC1, which belongs to the Shaker-like family is also inactive, but, unlike KCO3, remains located in the endoplasmic reticulum when overexpressed alone (Dreyer et al., 1997; Duby et al., 2008). Interaction of AtKC1 with other Shaker-like subunits allows the formation of traffic-competent, active heterotetramers with differences in activity compared to the respective homotetramers. AtKC1 thus acts as a modulator of channel activity. Furthermore, it has been shown that these heteromers can be further regulated by interaction with the SNARE protein SYP121 at the plasma membrane (Honsbein et al., 2009). KCO3 could in theory have a regulatory function by interacting with TPK subunits. Such an interaction could escape our assembly assay because of its transient or weak nature. Possibly, the formation of heteromers with KCO3 would prevent these TPK subunits from proper assembly into functional channels and expression of KCO3 would then have a modulatory function on certain tandem-pore channels. Although such a regulation does not appear to be very efficient, it might nevertheless be useful in fine-tuning the permeability of the tonoplast for K^+^ ions and would be consistent with the minor but detectable growth phenotype of the null allele that was complemented by a dominant-negative version of KCO3. However, it should be taken into consideration that, unlike AtKC1, overexpressed KCO3 traffics along the secretory pathway also in the absence of co-expressed putative partner subunits. Its tissue expression overlaps mostly with that of TPK5, but fluorescence transfer and bimolecular fluorescence complementation experiments failed to detect heteromeric interactions between KCO3 and TPK1 or TPK5 (Voelker et al., 2006). Heteromeric interactions seem therefore very unlikely, even if our results do not exclude that KCO3 actually forms a homotetramer by interactions that are not preserved in our conditions. Indeed, tetramers of Kcv, a viral K^+^ channel with one pore domain per subunit, are disrupted when K^+^ is substituted by Na^+^ or Li^+^ in the assay buffer. This disruption results in full Kcv disassembly into monomers, without the production of detectable dimers (Pagliuca et al., 2007). This supports the hypothesis that KCO3 dimers detected in the present study are stable, natural forms of this one P-subunit.

Recent fluorescence fluctuation analysis in transfected CHO cells suggested that human vanilloid receptor 1 (TRPV1), which belongs to the superfamily of cation channels with six transmembrane segments and one P-region, exists in two oligomerization states: dimeric in basal conditions and tetrameric upon activation (Storti et al., 2012). Similarly, it is possible that KCO3 dimers further assemble very transiently and reversibly or only upon certain physiological conditions: if only a very minor proportion of polypeptides were involved in this process at a given time, this could be below our detection limit. The process of further assembly could also require the presence of an additional factor. 14-3-3 proteins are among the candidates, since physical interaction and activity stimulation has also been shown for TPK1 (Latz et al., 2007).

In any case, our results indicate that KCO3 homodimers are as stable as TPK1 homodimers in the extraction and analysis conditions used here. If further assembly ever produces tetrameric KCO3 channels, at least part of the interactions involved must be different from those that hold together dimers. The search for such putative interactions would need further experimental approaches.

## Conflict of Interest Statement

The authors declare that the research was conducted in the absence of any commercial or financial relationships that could be construed as a potential conflict of interest.
